# Estimation of Nitrogen Status in *Zanthoxylum armatum* var. *novemfolius* Using Machine Learning Algorithms and UAV Hyperspectral and LiDAR Data Fusion

**DOI:** 10.3390/plants15071119

**Published:** 2026-04-06

**Authors:** Shangyuan Zhao, Yong Wei, Jinkun Zhao, Shuai Wang, Xin Ye, Xiaojun Shi, Jie Wang

**Affiliations:** 1 College of Resources and Environment, Southwest University, Chongqing 400716, China; zsy16635428084@email.swu.edu.cn (S.Z.); 17320128335@163.com (Y.W.); shixj@swu.edu.cn (X.S.); 2 Chongqing District Agricultural Technology Extension Center, Chongqing 401121, China; zzym9426@163.com (J.Z.); 3 National Agricultural Science Beibei Observation and Experiment Station, Chongqing 400716, China; 4 Chongqing Field Scientific Observatory of Soil Quality and Ecological Environment, Hechuan 401519, China

**Keywords:** nitrogen status estimation, hyperspectral imagery, LiDAR, data fusion, flight altitude optimization

## Abstract

Accurate monitoring of nitrogen (N) status is critical for precision N management and optimizing the yield and quality of *Zanthoxylum armatum* var. *novemfolius* (ZA). However, individual sensors often struggle to simultaneously capture the biochemical variations and complex canopy structural changes of ZA. Therefore, field experiments were conducted over two consecutive years, applying four N-application rates (0, 150, 300, and 450 kg N ha^−1^) to ZA. At each phenological stage, hyperspectral imagery and LiDAR point clouds were collected via three UAV flight altitudes (60 m, 80 m, and 100 m), and canopy nitrogen concentration (CNC) and aboveground nitrogen accumulation (AGNA) were measured. This study developed a framework by synergistically fusing UAV-derived hyperspectral imaging (HSI) and LiDAR data for CNC and AGNA monitoring. Results showed that the response of nitrogen status indicators to fertilization was phenology-specific: CNC showed no significant difference (*p* > 0.05) among treatments during the vigorous vegetative growth stage (VGS) but differed significantly (*p* < 0.05) during the fruit expansion stage (FES); AGNA differed significantly among treatments at VGS and FES (*p* < 0.05). The two-step screening yielded NDSI _(732, 879)_ and NDSI _(560, 690)_ as the optimal CNC indicators at VGS and FES, respectively (r = 0.83 and 0.93), whereas the NDSI _(711, 986)_ and NDSI _(515, 736)_ were identified as the optimal AGNA indicators at VGS and FES, respectively (r = 0.91 and 0.71). Across all phenological stages, Random Forest Regression consistently delivered the highest accuracy for CNC (R^2^ = 0.93–0.98, RMSE = 0.87–1.02 g kg^−1^) and AGNA (R^2^ = 0.95–0.97, RMSE = 1.92–2.55 g plant^−1^), outperforming MLR, PLSR, and SVR. This synergistic framework provides a high-precision, non-destructive methodology for the precision N monitoring of woody crops.

## 1. Introduction

*Zanthoxylum armatum* var. *novemfolius* (ZA), a characteristic economic woody spice in China, is widely cultivated for its unique aroma and medicinal properties [1]. Nitrogen (N) is one of the essential nutrient elements required for the growth of ZA [2]. A previous study has demonstrated that nitrogen status is the crucial diagnostic indicator for fruit development of ZA [3]. Precise N-monitoring techniques for the woody crop have been hindered by its complex canopy architecture and distinct phenological characteristics [4]. Previous studies have demonstrated that the phenological stages significantly influence nitrogen dynamics [5]. For instance, apple trees maintain higher N levels during flowering than during the fruit expansion stage [6]. Nevertheless, research investigating the interactive effects of N application rates and phenological stages on the nutritional status of ZA remains scarce. Furthermore, traditional diagnostic methods—primarily laboratory-based chemical analyses—are destructive, labor-intensive, and costly [7], necessitating the development of more efficient, non-destructive monitoring frameworks [8]. Currently, UAV-based hyperspectral imaging (HSI), which captures continuous narrow-band reflectance, offers significant advantages in quantifying nitrogen-related biochemical traits [6]. However, its application is often hindered by the “saturation effect” in dense perennial canopies [9]. This phenomenon occurs because HSI primarily captures surface reflectance, where upper leaves physically obscure the lower layers, effectively “blinding” the sensor to the biochemical information within the internal canopy volume once the leaf area reaches a certain density. To overcome this two-dimensional limitation, Light Detection and Ranging (LiDAR) is introduced to provide a critical three-dimensional perspective [10]. By penetrating the canopy to quantify structural parameters such as volume and density, LiDAR complements spectral data by accounting for the biomass depth that HSI fails to perceive, thereby significantly enhancing the accuracy of canopy nitrogen status estimation [11,12]. Furthermore, the optimization of UAV flight parameters is critical for accurate data acquisition, although the influence of flight altitude on nitrogen estimation has not been fully consistent across studies [13,14]. For instance, studies in citrus orchards have shown that lower altitudes (e.g., 50 m) provide richer structural detail but may introduce motion blur and increase data processing complexity, while higher altitudes (e.g., 120 m) may lead to the loss of small-scale canopy gaps essential for nitrogen estimation [4]. In cotton crops, research has demonstrated that as UAV flight altitude increases (e.g., from 60 m to 100 m), spectral reflectance in the visible range (500–550 nm) increases, while near-infrared reflectance decreases due to the inclusion of more soil background information in the pixels [14]. Determining the optimal flight altitude is therefore essential for improving data quality.

In this study, a multi-rotor UAV platform equipped with hyperspectral and LiDAR sensors was employed to collect data from a ZA orchard over two consecutive years. The specific objectives were to: (1) Analyze the variation in canopy nitrogen concentration (CNC) and aboveground nitrogen accumulation (AGNA) across the different phenological stages of ZA; (2) to develop a workflow for screening phenological sensitive UAV hyperspectral and LiDAR parameters for monitoring CNC and AGNA, including multiple flight altitudes (60, 80, and 100 m); (3) to develop highly accurate and robust models for CNC and AGNA monitoring by machine learning algorithms and UAV hyperspectral and LiDAR parameter fusion.

## 2. Results

### 2.1. Nitrogen Status Affected by Nitrogen Application Ratio and Different Stages

Both nitrogen application rates and growing stages significantly influenced the biomass, canopy nitrogen concentration, and aboveground nitrogen accumulation of *Zanthoxylum armatum* (ZA) (Table 1). During the vigorous vegetative growth stage (VGS), the nitrogen application ratio significantly influenced the biomass and aboveground nitrogen accumulation (AGNA) of ZA (*p* < 0.05), whereas the canopy nitrogen concentration (CNC) showed no significant differences among the nitrogen application treatments (*p* > 0.05), which is because of nitrogen dilution effect. Both biomass and AGNA exhibited a clear increasing trend with rising nitrogen application rates, reaching their peak values under the N3 treatment (2.73–4.04 kg plant^−1^; 37.12–49.15 g plant^−1^). However, the average CNC value among N1–N3 treatments was only 3.04% higher than that of the N0 treatment. During the fruit expansion stage (FES), the biomass showed no significant differences among the nitrogen application treatments (*p* > 0.05), whereas the CNC and AGNA showed significant differences among the nitrogen application treatments (*p* < 0.05). The CNC and AGNA exhibited a clear increasing trend with rising nitrogen application rates, reaching their peak values under the N2 treatment (35.41–39.76 g kg^−1^; 47.18–59.92 g plant^−1^). The yield of N2 was found to be significantly higher than the N0 and N1 treatments, with 5.24 kg plant^−1^ at Lishi town and 5.6 kg plant^−1^ at Ciyun town.

### 2.2. Correlations of Canopy Nitrogen Content and Above Ground Nitrogen Accumulation with UAV Hyperspectral and LiDAR Parameters Across Flight Altitudes and Growth Stages

**Figure 1:**
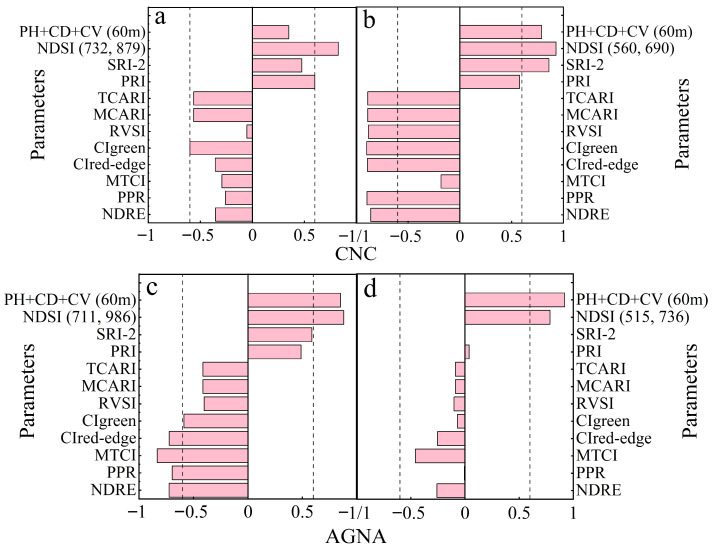
Pooled Spearman correlation analysis (2020–2021) of canopy nitrogen concentration (CNC) and aboveground nitrogen accumulation (AGNA) with hyperspectral vegetation indices and LiDAR structural parameters. Panels (**a**) and (**b**) represent CNC during the vigorous vegetative growth stage (VGS) and fruit expansion stage (FES), respectively, with hyperspectral data collected at a flight altitude of 100 m. Panels (**c**,**d**) represent AGNA during VGS and FES, where hyperspectral data were collected at 60 m for panel (**c**) and at 100 m for panel (**d**). For all scenarios, the LiDAR structural parameters were consistently derived from data collected at a flight altitude of 60 m.

The Spearman correlations of hyperspectral vegetation indices and LiDAR parameters with the canopy nitrogen concentration (CNC) and aboveground nitrogen accumulation (AGNA) also exhibited varying trends across different phenological stages (Figure 1). The correlation between LiDAR parameters (PH + CD + CV) and CNC reached 0.79 at the fruit expansion stage (FES), which was markedly higher than the correlation during the vigorous vegetative growth stage (VGS, r = 0.35). During the VGS, the correlations of 11 spectral parameters with CNC ranged from −0.05 to −0.36 (negative) and 0.47 to 0.83 (positive), with the correlation of NDSI _(732, 879)_ being the highest (0.83). At the FES, the correlations of 11 spectral parameters with CNC ranged from −0.18 to −0.90 (negative) and 0.58 to 0.93 (positive), among which the correlation of NDSI _(560, 690)_ was the highest (0.93). The direction (positive or negative) of these correlations is primarily determined by the mathematical structure of the index formulas. The correlations of LiDAR parameters (PH + CD + CV) with AGNA remained at a consistently high level, ranging from 0.85 to 0.92. During the VGS, the correlations of 11 spectral parameters with AGNA ranged from −0.41 to −0.76 (negative) and 0.45 to 0.91 (positive), with the correlation of NDSI _(711, 986)_ being the highest (0.91). At the FES, the correlations of 11 spectral parameters with CNC ranged from −0.01 to −0.46 (negative) and 0.04 to 0.78 (positive), among which the correlation of NDSI _(515, 736)_ was the highest (0.78). Furthermore, multicollinearity diagnostics and feature contribution analysis were conducted to verify model robustness and interpretability (Table S3).

### 2.3. Nitrogen Status Retrieval Models by Machine Learning Methods and Data Fusion

**Figure 2:**
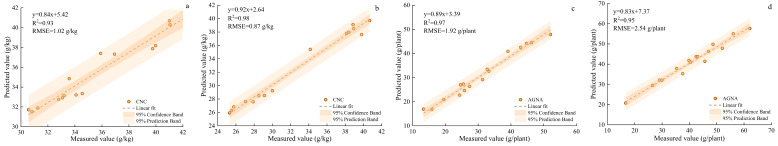
Comparison of measured and LOOCV-predicted (**a**,**b**) CNC and (**c**,**d**) AGNA using UAV-RFR models across phenological stages. Panels (**a**,**c**) represent VGS and (**b**,**d**) represent FES. Flight altitudes are 100 m for (**a**,**b**,**d**) and 60 m for (**c**). Model predictions were evaluated via LOOCV based on stage-specific datasets. For each phenological stage (VGS and FES), a sample size of *n* = 16 was utilized for model development and validation.

The predictive performance for CNC was significantly influenced by the regression models, with the coefficient of determination (R^2^) of Random Forest Regression (RFR, R^2^ = 0.93–0.98, RMSE = 0.87–1.02 g kg^−1^) always outperforming that of Multiple Linear Regression (MLR), Partial Least Squares Regression (PLSR), and Support Vector Regression (SVR) (R^2^ = 0.71–0.93, RMSE = 0.10–0.18 g kg^−1^) (Table 2). Similarly, the predictive performance for AGNA was significantly influenced by the regression models, with the R^2^ of Random Forest Regression (R^2^ = 0.95–0.97, RMSE = 1.92–2.55 g plant^−1^) always outperforming that of MLR, PLSR, and SVR (R^2^ = 0.80–0.93, RMSE = 0.21–0.52 g plant^−1^). The fitting relationship between the measured values and the predicted values obtained via the leave-one-out cross-validation method for CNC and AGNA using the RFR regression models across distinct phenological stages is displayed in Figure 2. Furthermore, our analysis of configuration trade-offs revealed that an Optimized Altitude (60 m LiDAR + 100 m HSI) achieved a 4.40–8.99% improvement in predictive accuracy compared to a uniform altitude (Table S4). While a uniform altitude simplifies flight planning, the Optimized Altitude approach maximizes the synergy between high-resolution structural metrics and noise-mitigated biochemical signals.

## 3. Discussion

### 3.1. Divergent Dominance of Pigment Sensitivity and Structural Biomass in Nitrogen Status Diagnostics Across Phenological Stages

Nitrogen in fruit trees exhibits a phase-transition trend across different phenological stages. To improve the accuracy of nitrogen monitoring, we investigate how vigorous growth stage (VGS) and fruit expansion stage (FES) affect nitrogen monitoring in fruit trees [15]. During the VGS of *Zanthoxylum armatum* (ZA), the elongating canopy and rapidly expanding leaf area require a sustained, adequate N supply to drive chlorophyll synthesis; yet this supply must remain balanced—excess N triggers uneven partitioning between flowers and leaves, while the N that is taken up is continuously channeled into photosynthetic production that fuels new leaf growth. During VGS, our results show that canopy nitrogen concentration (CNC) remained virtually flat, whereas aboveground nitrogen accumulation (AGNA) responded sharply to incremental nitrogen supply. During the VGS, a study of ZA revealed an equivalent split-response: CNC in leaves and fruits shifted by only 21.11% and 11.22% across fertilizer levels, whereas nitrogen accumulation AGNA extended across 53.84% and 54.31% [2]. CNC prediction performed better during FES than VGS. This is due to the nitrogen dilution effect, where rapid structural dry matter accumulation dilutes nitrogen concentration as trees develop [16,17]. Furthermore, AGNA correlate more strongly with LiDAR structural parameters, because it integrates both nitrogen concentration and total biomass. LiDAR effectively captures three-dimensional canopy structures (e.g., volume and height), which directly reflect the biomass allocation dynamics [18]. Consequently, the pronounced phase-transition of Nitrogen status between VGS and FES generates corresponding, fine-scale variations in canopy reflectance that conventional fixed-band indices often miss. The superior performance of the reconstructed Normalized Difference Spectral Index (NDSI) across all flight altitudes and growth stages confirms that full-spectrum optimization (400–1000 nm) is critical for overcoming signal saturation in dense perennial canopies [19]. By dynamically identifying optimal band pairs, the NDSI framework captures subtle nitrogen-induced shifts that fixed-band indices often fail to detect [20]. Furthermore, our findings reveal a significant phenological link between the optimal spectral regions and the physiological priorities of the crop: during the VGS, the optimal NDSI band pairs (e.g., 711, 732, and 986 nm) shifted toward the 700–1000 nm near-infrared region, which is highly indicative of plant biomass and internal leaf cell structure [21]. As VGS is characterized by rapid dry matter accumulation and structural expansion, these NIR signals reflect the plant’s physical volume and density, aligning with the retrieval requirements for AGNA. Conversely, during the FES, the optimal band pairs (e.g., 515, 560, and 690 nm) were consistently located within the 400–700 nm visible light range, a region dominated by the absorption of photosynthetic pigments such as chlorophyll and carotenoids [22]. The massive translocation of nitrogen from leaves to fruits during this stage triggers sensitive fluctuations in pigment concentration, making the visible spectrum the most potent window for diagnosing CNC. To leverage these spectral sensitivities, this framework integrates hyperspectral-based chemical analysis with LiDAR-derived structural mapping, thereby enabling a more comprehensive assessment of nitrogen status by combining nitrogen concentration data with total biomass estimates. This approach ensures that both nutrient intensity and total nitrogen content are accurately quantified within the complex spatial architecture of the orchard [18].

### 3.2. Synergistic Effects of Multi-Source Data Fusion and Machine Learning

The integration of hyperspectral imagery and LiDAR point clouds demonstrated a superior capacity for capturing the complex nitrogen (N) dynamics of *Zanthoxylum armatum* (ZA) compared to single-sensor approaches. In this study, the Random Forest Regression (RFR) model consistently outperformed traditional methods like MLR and PLSR, achieving a peak R^2^ of 0.98. The superior performance of RFR over SVR (R^2^: 0.78–0.93) stems from its inherent advantage in managing the highly non-linear, high-dimensional data typical of dense canopies, whereas, SVR relies on a global optimal hyperplane that is often overly sensitive to hyperparameter tuning in heterogeneous datasets [23]. Similar robust performances of RFR in multi-source fusion have been documented in other woody crops, such as almond orchards, where the inclusion of structural variables significantly refined canopy-scale N estimations [18]. The synergy between the two sensors effectively addresses the “saturation effect” that often limits the precision of passive optical sensors in dense perennial canopies. While optimized hyperspectral indices (e.g., NDSI) are highly sensitive to N-induced chlorophyll shifts in the upper canopy layer, they frequently fail to reflect the N status of the sub-canopy. By incorporating LiDAR-derived variables—specifically the PH + CD + CV triplet—the model accounts for the “physical” context of the 3D architecture, which is a critical predictor for nitrogen accumulation (AGNA). This aligns with recent findings that integrating canopy height and volume data into spectral models provides a more complete picture of nitrogen status by combining LiDAR-derived structure and hyperspectral-derived chemistry [13].

### 3.3. Impact of UAV Flight Altitude on Retrieval Precision

A critical finding of this research is that the “optimal” UAV flight altitude is not universal but is highly dependent on both the target nitrogen trait and the specific growth stage. For canopy nitrogen concentration (CNC) retrieval, a higher altitude of 100 m generally yielded the best performance, with fusion data reaching peak R^2^ values during both growth stages. At increased heights, the sensor’s larger footprint effectively averages out “leaf-level” noise, such as localized specular reflection and shadow occlusion within the canopy [13,24]. By contrast, Aboveground nitrogen accumulation (AGNA) retrieval responded differently to flight height depending on phenological stage. During FES the 100 m altitude again delivered the highest fitting accuracy (R^2^ = 0.95), yet in VGS the 60 m flight level outperformed both the 80 m and 100 m, boosting fitting accuracy by 2.95–3.27%. We attribute this switch to the biomass-oriented band pair selected by the reconstructed NDSI at 60 m (711 and 986 nm); at higher altitudes the same index shifted toward shorter wavelengths that are less sensitive to canopy volume, thereby weakening the correlation with AGNA [1,20]. For the LiDAR component, 60 m was consistently superior for both growth stages and both nitrogen traits (Table S2). The 60 m flight altitude provides significantly higher point cloud density, enabling the precise quantification of canopy physical parameters like height and volume [25]. This physical precision improves AGNA retrieval by providing a more accurate biomass basis for mass-based estimation, while also helping CNC models account for shadowing and canopy heterogeneity [26]. Collectively, the results prescribe a flight plan that is sensor-specific rather than universal: 100 m for hyperspectral acquisition to maximize biochemical signal-to-noise, 60 m for LiDAR to preserve the structural fidelity required for biomass-linked nitrogen mapping.

### 3.4. Limitations and Future Perspectives

While the integration of UAV-derived HSI and LiDAR data demonstrated significant potential for nitrogen monitoring, several limitations should be acknowledged. First, the sample size in this study was relatively limited (*n* = 16 per stage), which is a common challenge in research involving perennial woody crops due to the high costs and the destructive sampling (over 500 yuan per tree). Thus, we ultimately used leave-one-out cross-validation to build the model. Although leave-one-out cross-validation was utilized to maximize the utility of the available data, but it may tend to overestimate performance. Second, due to the limited total number of samples (*n* = 32), this study did not implement a fully independent validation set from different years or varieties. In future work, the dataset should be expanded to include a larger sample size, which can then be divided into training, validation, and test sets to further verify the generalizability and robustness of the diagnostic framework. Third, regarding the modelling methods, this study primarily evaluated traditional machine learning algorithms. While Deep Learning (DL) architectures offer powerful capabilities for multi-modal data fusion, modern deep learning architectures (e.g., convolutional neural networks for spectral–spatial cubes, pointNet for point clouds or transformer-based spectral models) could potentially capture non-linear canopy–nitrogen relationships more effectively than ensemble trees. Although our current dataset is limited, future research should leverage these DL frameworks to enhance modeling performance as larger datasets become available. Finally, external environmental factors such as wind speed, complex terrain, and orchard spacing were not fully integrated into the current model. Future work should incorporate these practical constraints and environmental variables to enhance the operational feasibility of the framework in diverse and complex orchard environments.

## 4. Materials and Methods

### 4.1. Study Site and Experimental Design

**Figure 3:**
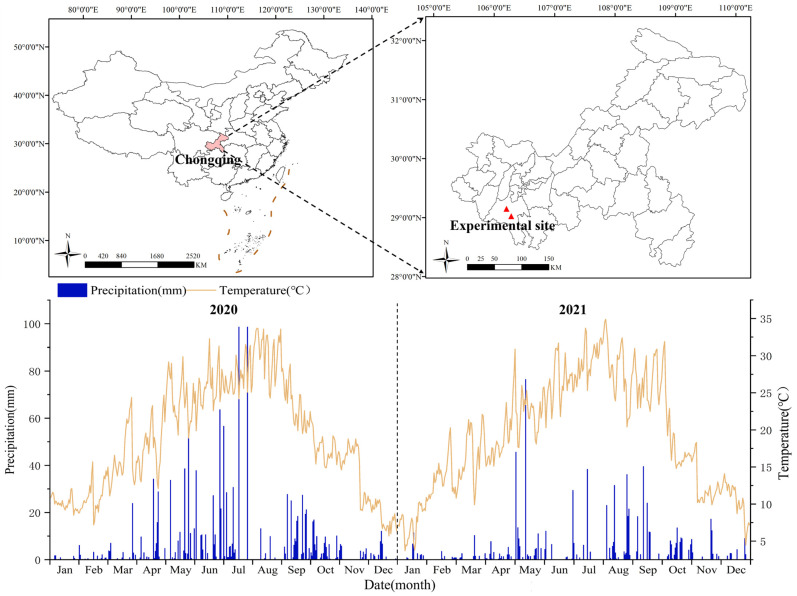
Study area location and the variations of temperature and precipitation in 2020 and 2021. The red triangle indicates the location of the experimental site.

The study was conducted from 2020 to 2021 at the cultivation bases of Lishi Town and Ciyun Town, Chongqing, China (106.1800° E, 29.0700° N, altitude 330 m) (Figure 3). These two orchards were selected as independent experimental sites because they represent the two most typical large-scale commercial cultivation models in the region. Specifically, the Ciyun site is a high-yield demonstration base characterized by intensive precision management, while the Lishi site follows traditional large-scale farming practices with moderate productivity levels. The study region is characterized by an annual average temperature of 18.2 °C and an annual average precipitation of 1034.7 mm, which belongs to a subtropical humid monsoon climate (Figure 1). The cultivars used in this experiment were the primary *Zanthoxylum armatum* (ZA) grown in Jiangjin, Chongqing. These 8-year-old trees have been in continuous fruit production for over 5 years, exhibit strong adaptability to the local climate, and are recognized for their stable performance These characteristics ensure that the selected ZA are both representative and suitable for evaluating canopy nitrogen concentration (CNC) and aboveground nitrogen accumulation (AGNA) under different nitrogen concentration.

The fertilizer treatments and soil properties are summarized in Table 3. This experiment included four N application rates: N0 (0 kg N ha^−1^, control), N1 (150 kg N ha^−1^), N2 (300 kg N ha^−1^, the local recommended rate), and N3 (450 kg N ha^−1^, the conventional rate used by local farmers) [2]. Nitrogen was supplied as urea (CO(NH_2_)_2_, 46% N). Phosphorus and potassium were applied at total rates of 120 kg P_2_O_5_ ha^−1^ and 100 kg KCl ha^−1^, respectively. Phosphorus and potassium were supplied as calcium superphosphate, 12% P_2_O_5_ and muriate of potash, 62%. These treatments were arranged in a randomized complete block design with three replicates, with each replicate comprising 10 individual trees to ensure representative sampling. Fertilizers were applied in four phenological stages: shoot-promoting (June), autumn basal (late September), bud break (next February), and fruit expansion (next April). These nutrients were partitioned across the four application timings according to the following proportions: nitrogen (30%, 30%, 20%, and 20%), phosphorus (50%, 20%, 20%, and 10%), and potassium (30%, 10%, 20%, and 40%), respectively. The planting density was 1667 plants per hectare, with a row spacing of 2 m and a plant spacing of 3 m. The predominant soil type in the area is purple soil. The soil nutrient backgrounds (Table 3) provide different alkaline nitrogen. Adequate irrigation and pest management ensured minimal stress during crop growth.

### 4.2. The Framework of This Study

**Figure 4:**
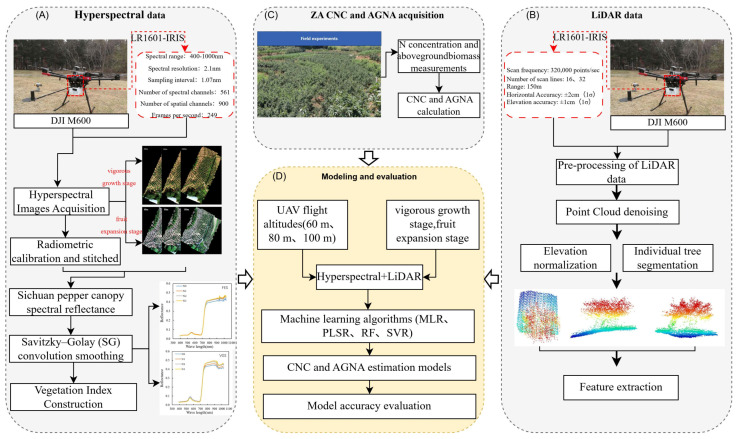
The technical diagram of this study. (**A**) UAV hyperspectral data processing; (**B**) UAV LiDAR data processing; (**C**) field destructive data collection; (**D**) model construction and evaluation. The color scale from blue to red represents the transition from low to high point cloud density.

The study workflow (Figure 4) utilizes a multi-source UAV platform for simultaneously acquisition of hyperspectral and LiDAR data. Two-year experiments (2020–2021) provided data for constructing *Zanthoxylum armatum* (ZA) canopy nitrogen concentration (CNC) and aboveground nitrogen accumulation (AGNA) estimation models at the vigorous vegetative and fruit-expansion stages. Machine learning algorithms (MLR, PLSR, SVM, and RFR) were applied to different flight altitude combinations (60 m, 80 m, and 100 m) to develop these models. Factors influencing model estimation precision were thoroughly examined, with particular attention paid to the growth stage, flight height, and algorithm performance impacts.

### 4.3. Ground Data Acquisition

Prior to remote-sensing data collection, two *Zanthoxylum armatum* (ZA) trees with uniform growth statuses were selected and marked. Immediately after the remote sensing data were collected, destructive aboveground sampling was conducted on the target ZA trees. Sixteen trees were sampled during each growth stage (vigorous vegetative growth and fruit expansion stages), resulting in a total of 32 sample trees. The specific sampling method was as follows: the trunk was cut off near the ground level, and the aboveground parts were separated into four sections: branches, leaves, fruits, and main stem. The fresh weights of each part were measured. For each section, approximately 200 g of samples were collected from the east, west, south, and north sides of the canopy respectively. Of these, 100 g was placed in a self-sealing bag and stored in a refrigerator for further measurement; the remaining 100 g was thoroughly washed with deionized water, air-dried, weighed for fresh weight, then subjected to enzyme inactivation at 105 °C for 30 min in an oven (Shanghai Yiheng Scientific Instrument Co., Ltd., Shanghai, China), and dried at 65 °C to constant weight before being weighed and recorded. The dried samples were ground, passed through a 60-mesh sieve, and used for nitrogen content determination. The samples were digested using the concentrated H_2_SO_4_-H_2_O_2_ (Sinopharm Chemical Reagent Co., Ltd., Shanghai, China) method, and the total nitrogen content was measured using a Kjeldahl apparatus (Hanon Advanced Technology Group Co., Ltd., Jinan, China) [27].

CNC = NC_leaf_ (1)

AGNA = NC_leaf_ × M_leaf_ + NC_shoot_ × M_shoot_ + NC_fruit_ × M_fruit_ + NC_trunk_ × M_fruit_ (2)

where CNC = canopy nitrogen concentration (g kg^−1^); AGNA = above-ground nitrogen accumulation (g plant^−1^); NC_leaf_, NC_shoot_, NC_fruit_, NC_trunk_ = nitrogen concentration in leaf, shoot, fruit, and trunk, respectively (g kg^−1^); M_leaf_, M_shoot_, M_fruit_, M_trunk_ = dry biomass of leaf, shoot, fruit, and trunk, respectively (g plant^−1^).

### 4.4. UAV Data Acquisition and Processing

#### 4.4.1. UAV Platform

In this study, a DJI M600 Pro hexacopter UAV (DJI Company, Shenzhen, China) equipped with an IRIS integrated hyperspectral-LiDAR imaging system (LICA United Technology Limited, Beijing, China) was used to simultaneously collect hyperspectral imagery and LiDAR point cloud data of the *Zanthoxylum armatum* (ZA) canopy. Table 4 outlines the specifications of the IRIS integrated hyperspectral-LiDAR imaging system. To ensure the quality of the hyperspectral images and point cloud stitching, the UAV was programmed with an 80% forward overlap and a 60% side overlap, maintaining a flight speed of 1 m s^−1^ at altitudes of 60 m, 80 m, and 100 m. Each acquisition was conducted under clear and cloudless conditions between 11:00 a.m. and 2:00 p.m. Furthermore, to cover the growth characteristics of ZA during its key developmental stages, the above-mentioned remote sensing data were collected in the experimental areas of Lishi Town and Ciyun Town during late October (the end of the vigorous vegetative growth stage, VGS) and mid-April (the end of the fruit-expansion stage, FES) of both 2020 and 2021, respectively.

#### 4.4.2. Hyperspectral Image and LiDAR Data Processing

Hyperspectral images were collected and initially stitched using Agisoft PhotoScan to generate digital ortho-reflectance images. To ensure the positional accuracy of the hyperspectral image, nine pieces of 60 cm × 60 cm white reference plates were evenly placed around and in the center of the sample orchard as control points, and their geographical coordinates were recorded for geometric correction. The corrected images were radiometrically calibrated using deployed PTFE standard gray panels to convert the image’s Digital Number (DN) values into the reflectance of the *Zanthoxylum armatum* (ZA) canopy. Regions of Interest (ROIs) for the target ZA canopy were constructed based on marked information (selecting 50 pixels in each cardinal direction within the canopy while avoiding soil, ground vegetation, and shaded areas), and the average reflectance was extracted as the spectral reflectance for each sample point. Finally, the obtained hyperspectral data were subjected to noise reduction using the Savitzky–Golay (SG) convolution smoothing algorithm, resulting in canopy reflectance curves for modeling analysis. Simultaneously, Raw point clouds were pre-processed in LiDAR360 (version 5.0, GreenValley International, Berkeley, CA, USA) for noise removal and ground classification via the Progressive Densification TIN filtering algorithm. A smoothed Canopy Maxima Model was then generated to facilitate individual tree segmentation using a marker-controlled watershed algorithm. Finally, structural parameters were extracted using PCM software (version 2.0, Aerospace Information Research Institute, Chinese Academy of Sciences, Beijing, China): Plant Height and Crown Diameter were derived from the maximum vertical height and the average of cardinal crown widths, respectively, while Canopy Volume was quantified using the 3D convex hull method to accurately characterize the ZA canopy architecture [23]. These features were then used for machine learning modeling; specifically, the Random Forest Regression model was constructed with 100 decision trees, a minimum samples split of 2, a minimum samples leaf of 2, and a maximum depth of 3 [28,29]. A leave-one-out cross-validation strategy was employed to evaluate model stability. Additionally, Variance Inflation Factor (VIF) analysis and SHAP-based feature attribution were conducted to evaluate multi-collinearity and variable importance, respectively.

### 4.5. Modeling Methods

#### 4.5.1. Feature Selection

**Figure 5:**
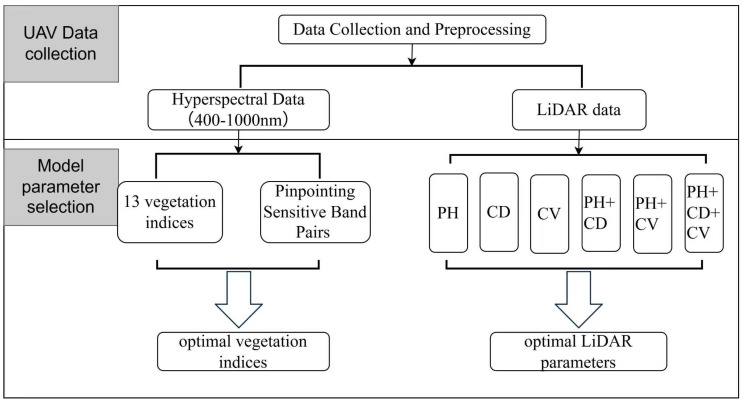
Flowchart of the optimized feature extraction and fusion process for CNC and AGNA estimation.

We implemented a comprehensive two-step sequential feature screening and optimization workflow (Figure 5) to identify the most robust predictors for canopy nitrogen concentration (CNC) and aboveground nitrogen accumulation (AGNA) in *Zanthoxylum armatum* (ZA). Initially, the regions with the coefficient of determination (R^2^) values for NDSI, CNC, and AGNA are calculated from 400–1000 nm when the UAV flight altitude is 60–100 m (Figures S1 and S2). Secondly, the correlation coefficient between nitrogen status and LiDAR based characteristic parameters is calculated when the UAV flight altitude is 60–100 m (Table S2).

#### 4.5.2. Machine Learning

To predict the CNC and AGNA contents in *Zanthoxylum armatum* (ZA), four machine learning algorithms were evaluated: Multiple Linear Regression (MLR), Partial Least Squares Regression (PLSR), Support Vector Regression (SVR), and Random Forest Regression (RFR). To enhance the spatiotemporal robustness of the models, data from different years (2020 and 2021) and locations (Lishi and Ciyun) were pooled into a single dataset for analysis. Conversely, separate models were constructed for each growth stage (vigorous growth stage and fruit expansion stage) and each UAV flight altitude (60 m, 80 m, and 100 m) to evaluate stage-specific physiological fluctuations and the impact of observation height on diagnostic accuracy.

Given the limited sample size (*n* = 16 per phenological stage), leave-one-out cross-validation was adopted to maximize data utility and ensure unbiased estimation. Furthermore, individual trees within the same treatment block or spatially adjacent trees could appear in both training and validation folds during LOOCV. This iterative procedure reserves each observation as a validation set once while training the model on the remaining n-1 samples; the resulting performance metrics (R^2^ and RMSE) thus represent the aggregate results across all cross-validation iterations. Specifically, MLR was used to model linear dependencies, while PLSR was selected for its ability to manage high-dimensional, collinear spectral data through latent variable projection [30]. For non-linear modeling, RFR was utilized for its ensemble-based robustness against noise, While SVR was employed to define a maximum-margin hyperplane using the loss function [31,32].

#### 4.5.3. Model Accuracy Evaluation

Statistical analysis was performed using various metrics to evaluate the performance of the model. The coefficient of determination (R^2^), Root Mean Square Error (RMSE), and Mean Relative Error (MRE) were used to assess the model. An R^2^ value closer to 1 indicates a better estimation performance by the model. Lower RMSE and MRE values also indicate better estimation performance. The formulas for these metrics are as follows:

(3) $$R^{2}=\sum_{i=1}^{n}{\left({\hat{y}}_{i}-\bar{y}\right)}^{2}/\sum_{i=1}^{n}{\left(y_{i}-\bar{y}\right)}^{2}$$

(4) $$RMSE=\sqrt{\sum_{i=1}^{n}{\left({\hat{y}}_{i}-y_{i}\right)}^{2}/n}$$

(5) $$MRE=\frac{1}{n}\sum_{i=1}^{n}\frac{\left|y_{i}-{\hat{y}}_{i}\right|}{y_{i}}$$

where $y_{i}$ represents the observed value, $\bar{y}$ represents the mean of the observed values, ${\hat{y}}_{i}$ represents the predicted value, i represents the sample index, and n represents the number of samples.

#### 4.5.4. Statistical Analysis

Spectral data were processed using ENVI 5.3 (L3Harris Geospatial, Boulder, CO, USA), point cloud data were processed using LiDAR360 and PCM software. Vegetation indices were constructed using Python 3.1.4. Statistical differences in biomass, CNC, and AGNA among the nitrogen treatments were evaluated using one-way analysis of variance (ANOVA) followed by Tukey’s post-hoc test at a significance level of *p* < 0.05. All models were implemented in Python (v3.8) using the scikit-learn library (v0.24.2). Graphs were plotted using Origin 2024 (OriginLab Corporation, Northampton, MA, USA).

## 5. Conclusions

Stage-specific fusion of UAV hyperspectral and LiDAR data, refined by a two-step feature selector and Random Forest regression, delivered CNC R^2^ = 0.93–0.98 (RMSE 0.87–1.02 g kg^−1^) and AGNA R^2^ = 0.95–0.97 (RMSE 1.92–2.55 g plant^−1^) across VGS and FES, providing a robust, non-destructive nitrogen monitoring tool for precision management of *Zanthoxylum armatum* and other woody crops.

## Figures and Tables

**Table 1:** Statistical analysis (mean ± SD; ANOVA post hoc Tukey) of biomass, canopy nitrogen concentration (CNC), aboveground nitrogen accumulation (AGNA), and yield of *Zanthoxylum armatum* var. *novemfolius* under different nitrogen application rate treatments (N0, N1, N2, and N3) at the Vigorous vegetative growth stage (VGS) and the fruit expansion stage (FES). Different lowercase letters within a row indicate significant differences among treatments at *p* < 0.05. The significant variables were colored blue ranging from the lowest (light blue) to the highest (dark blue) values.

		Lishi					Ciyun				
		N0	N1	N2	N3	*p*-Value	N0	N1	N2	N3	*p*-Value
VGS	Biomass (kg plant^−1^)	1.38 ± 0.05 c	1.86 ± 0.15 b	1.91 ± 0.18 b	2.73 ± 0.16 a	0.004	2.81 ± 0.12 c	3.18 ± 0.09 b	3.89 ± 0.05 a	4.08 ± 0.10 a	<0.001
	CNC (g kg^−1^)	38.06 ± 0.73 a	38.86 ± 1.25 a	39.35 ± 1.06 a	39.95 ± 1.38 a	0.477	32.19 ± 0.53 a	32.48 ± 0.39 a	33.02 ± 0.33 a	33.58 ± 0.72 a	0.172
	AGNA (g plant^−1^)	17.70 ± 2.58 c	24.48 ± 0.21 b	26.61 ± 1.10 b	37.12 ± 0.76 a	<0.001	24.44 ± 1.52 d	32.06 ± 1.00 c	40.53 ± 1.75 b	49.15 ± 4.10 a	0.037
FES	Biomass (kg plant^−1^)	2.58 ± 0.12 a	2.69 ± 0.16 a	2.81 ± 0.12 a	2.87 ± 0.18 a	0.330	3.68 ± 0.18 a	3.74 ± 0.10 a	3.98 ± 0.18 a	4.12 ± 0.32 a	0.277
	CNC (g kg^−1^)	32.58 ± 0.31 c	35.62 ± 0.63 b	39.76 ± 1.28 a	38.54 ± 0.56 a	0.003	22.63 ± 1.23 d	28.00 ± 1.24 c	35.41 ± 1.26 a	32.35 ± 1.11 b	0.012
	AGNA (g plant^−1^)	32.65 ± 3.80 c	38.24 ± 0.98 bc	47.18 ± 2.30 a	41.63 ± 1.75 ab	0.131	32.89 ± 1.15 d	40.15 ± 3.24 c	59.92 ± 3.09 a	51.51 ± 3.22 b	0.002
Yield (kg plant^−1^)		3.08 ± 0.53 c	3.77 ± 0.48 bc	5.24 ± 0.45 a	4.35 ± 0.54 ab	0.004	3.27 ± 0.58 c	3.92 ± 0.30 c	5.61 ± 0.26 a	4.76 ± 0.55 b	0.001

Note: Different lowercase letters in columns indicate significant differences between different nitrogen application treatments (*p* < 0.05). VGS represents the vigorous vegetative growth stage, which is primarily the structural development stage of the tree, involving the rapid expansion of leaves and the elongation of new shoots. FES represents the fruit expansion stage, which is characterized by the rapid growth and volume increase of the *Zanthoxylum armatum* fruit.

**Table 2:** Performance comparison of machine learning algorithms for canopy nitrogen concentration (CNC) and above-ground nitrogen accumulation (AGNA) estimation based on optimal feature fusion (LiDAR + Hyperspectral) across phenological stages and leave-one-out cross-validation (LOOCV).

	**Phenological Stage**	**Parameters**		**PLSR**		**MLR**		**SVR**		**RFR**	
		PH + CD + CV *	NDSI	R^2^	RMSE	R^2^	RMSE	R^2^	RMSE	R^2^	RMSE
CNC (g kg^−1^)	VGS		732, 879 #	0.71 ± 0.08	2.09 ± 0.18	0.72 ± 0.08	2.05 ± 0.17	0.78 ± 0.06	1.81 ± 0.15	0.93 ± 0.02	1.02 ± 0.09
	FES		560, 690 #	0.86 ± 0.04	2.16 ± 0.11	0.87 ± 0.04	2.05 ± 0.10	0.93 ± 0.02	1.48 ± 0.06	0.98 ± 0.01	0.87 ± 0.05
AGNA (g plant^−1^)	VGS		711, 986 †	0.89 ± 0.51	4.76 ± 0.29	0.91 ± 0.03	3.36 ± 0.21	0.93 ± 0.02	4.71 ± 0.31	0.97 ± 0.01	1.92 ± 0.18
	FES		515, 736 #	0.87 ± 0.04	4.12 ± 0.25	0.88 ± 0.04	3.92 ± 0.24	0.80 ± 0.06	8.38 ± 0.52	0.95 ± 0.02	2.55 ± 0.22

Note: VGS and FES represent the vigorous vegetative growth stage and fruit expansion stage, respectively. Accuracy metrics (R^2^ and RMSE) were calculated via LOOCV based on stage-specific datasets. For each phenological stage (VGS and FES), a sample size of *n* = 16 was utilized for model development and LOOCV. The symbols in the table indicate the specific UAV flight altitudes at which the corresponding parameters were acquired: *: 60 m, #: 100 m, and †: 60 m. MLR, PLSR, SVR, and RFR represent the machine learning algorithms evaluated for nitrogen status estimation. Model performance is quantified by R^2^ (coefficient of determination) and RMSE (root mean square error; expressed in g kg^−1^ for CNC and g plant^−1^ for AGNA).

**Table 3:** Basic information regarding two field experiments.

Experimental Site	Year	Treatments	Soil Characteristics
Lishi Town	2020–2021	N application (kg N ha^−1^): 0, 150, 300, 450	pH: 4.4 Organic matter: 12.06 g kg^−1^ Alkaline-N: 174.20 mg kg^−1^ Olsen-P: 46.16 mg kg^−1^ Available-K: 232 mg kg^−1^
Ciyun Town	2020–2021	N application (kg N ha^−1^): 0, 150, 300, 450	pH: 4.9 Organic matter: 14.38 g kg^−1^ Alkaline-N: 78.65 mg kg^−1^ Olsen-P: 21.49 mg kg^−1^ Available-K: 224 mg kg^−1^

**Table 4:** Specifications of the IRIS Integrated Hyperspectral LiDAR Imaging System.

Host Platform	DJI M600 Multi-Rotor Drone	
Support system	Multiple GNSS systems including BeiDou, GPS, GLONASS, and RTK	
Hyperspectral	Spectral range	400–1000 nm
	Spectral resolution	2.1 nm
	Sampling interval	1.07 nm
	Number of spectral channels	561
	Number of spatial channels	900
	Frames per second	249
LiDAR	Scan frequency	320,000 points s^−1^
	Number of scan lines	16, 32
	Range	150 m
	Horizontal Accuracy	±2 cm (1σ)
	Elevation accuracy	±1 cm (1σ)

## Data Availability

The original contributions presented in this study are included in the article/Supplementary Material. Further inquiries can be directed to the corresponding author.

## Supplementary Materials

The following supporting information can be downloaded at: https://www.mdpi.com/article/10.3390/plants15071119/s1, Table S1: Vegetation Indices, LiDAR induces and their corresponding descriptions and references; Table S2: correlation coefficient between nitrogen status and LiDAR based characteristic parameters; Table S3: Multicollinearity diagnostics (VIF) and feature importance analysis (SHAP) for the optimal LiDAR–HSI fusion models across phenological stages; Table S4. Comparison of model performance (R^2^) under Uniform Altitude and Optimized Altitude for CNC and AGNA estimation; Figure S1: Equipotential diagram of the correlation between any two spectral indices at different heights and the CNC of *Zanthoxylum armatum*; Figure S2: Equipotential diagram of the correlation between any two spectral indices at different heights and the AGNA of *Zanthoxylum armatum*. References [33,34,35,36,37,38,39,40] are cited in Supplementary Materials.
