# Supplementary material for: Estimation of Nitrogen Status in Zanthoxylum armatum var. novemfolius Using Machine Learning Algorithms and UAV Hyperspectral and LiDAR Data Fusion
Source: Plants (Basel). 2026 Apr 6;15(7):1119. doi: 10.3390/plants15071119 (PMC13075218; doi:10.3390/plants15071119)
Supplement: Supplementary file 1 [file plants-15-01119-s001.zip › plants-4139724-supplementary.pdf]

Supplementary Materials

Table S1 lists the 11 hyperspectral vegetation indices and 3 LiDAR-derived structural parameters used for nitrogen estimation in *Zanthoxylum armatum*. The vegetation indices include pigment-sensitive metrics (TCARI, MCARI, CI_{green}, and PPR) designed to isolate biochemical signals by minimizing interference from soil background and tree structure, as well as red-edge and broadband indicators (NDRE, MTCI, RVSI, and $CI_{red\text{-}edge}$) that monitor nitrogen concentration and photosynthetic capacity by leveraging the red-edge region to overcome signal saturation in dense foliage. Additionally, metabolic and optimized indices such as PRI, NDSI, and SRI-2 assess light-use efficiency and maximize spectral sensitivity to nitrogen chemistry through optimized band-pairing across different growth stages. The LiDAR triplet—plant height (PH), crown diameter (CD), and canopy volume (CV)—quantifies 3-D architecture.

Table S1. Vegetation Indices, LiDAR induces and their corresponding descriptions and references.

Variable	Description	Reference
NDRE	$(R_{700}-R_{710})/(R_{700}+R_{710})$	Zhou et al., 2019
PPR	$(R_{550}-R_{450})/(R_{550}+_{450})$	Wang et al., 2004
MTCI	$(R_{700}-R_{710})/(R_{710}-R_{670})$	Yin et al., 2021
$CI_{red\text{-}edge}$	$(R_{700}/R_{710})-1$	Gitelson, 2005
CI_{green}	$(R_{700}/R_{550})-1$	Gitelson, 2005
TCARI	$3\times[(R_{700}-R_{670})-0.2\times(R_{700}-R_{550})](R_{700}/R_{670})$	Haboudane et al., 2002
PRI	$(R_{531}-R_{570})/(R_{531}+R_{570})$	Gamon et al., 1992
RVSI	$((R_{712}-R_{753})/2)\text{-}R_{733})$	Almeida et al., 2021
MACRI	$[(R_{700}-R_{670})-0.2\times(R_{700}-R_{550})](R_{700}/R_{670})$	Daughtry et al., 2000
SRI-2	R_{515}/R_{570}	Hernández-Clemente et al., 2012
NDSI	$(R_i\text{-}R_j)/(R_i+R_j)$	Zhou et al., 2019
PH	Maximum Z-value of the segmented point cloud	Gao et al., 2022b
CD	X/Y-direction max-min difference	Gao et al., 2022b
CV	Point cloud volume	Gao et al., 2022b

In the vigorous vegetative growth stage (VGS), the regions with the coefficient of determination (R^2) values for NDSI and CNC are primarily concentrated around 680-1000 nm when the UAV flight altitude is 60-100 m (Fig S1). Specifically, the R^2 between the $NDSI_{(879,\ 732)}$ reconstructed from hyperspectral images at 100 m altitude and CNC is 0.68. During the fruit expansion stage (FES), the regions with larger R^2 values for NDSI and CNC are primarily concentrated around 520-680 nm when the UAV flight altitude is 60-100 m (Fig. S1). Specifically, the R^2 between the $NDSI_{(560,\ 690)}$ reconstructed from hyperspectral images at 100 m altitude and CNC is 0.88.

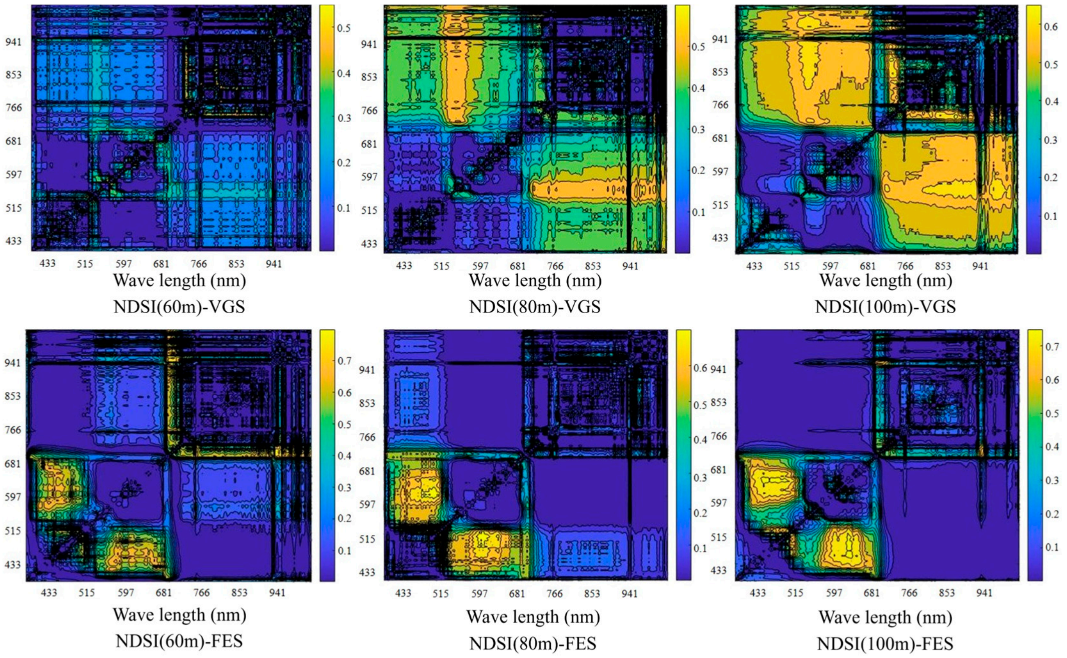

Figure S1. Equipotential diagram of the correlation between any two spectral indices at different heights and the CNC of *Zanthoxylum armatum*

In the vigorous vegetative growth stage (VGS), the regions with the coefficient of determination (R^2) values for NDSI and AGNA are primarily concentrated around 450-680 nm and 700-1000 nm when the UAV flight altitude is 60-100 m (Fig S2). Specifically, the R^2 between the NDSI $_{(986,\ 711)}$ reconstructed from hyperspectral images at 100 m altitude and AGNA is 0.83. During the fruit expansion stage (FES), the regions with larger R^2 values for NDSI and AGNA are primarily concentrated around 510-1000 nm when the UAV flight altitude is 60-100 m (Fig. S2). Specifically, the R^2 between the NDSI $_{(736,\ 515)}$ reconstructed from hyperspectral images at 100 m altitude and AGNA is 0.62.

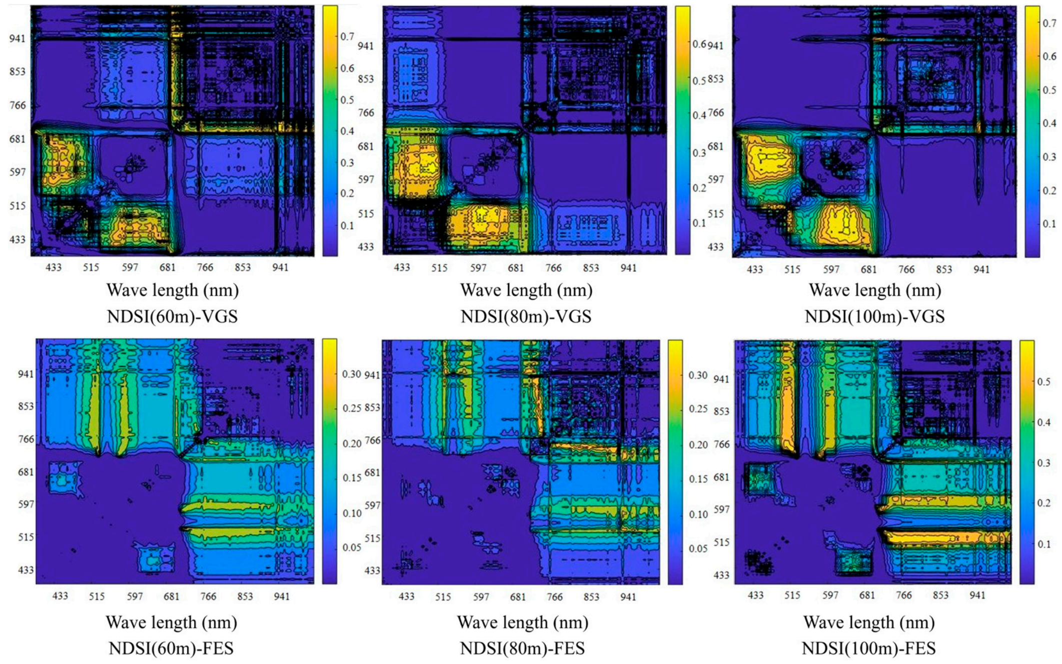

Figure S2. Equipotential diagram of the correlation between any two spectral indices at different heights and the AGNA of *Zanthoxylum armatum*

The PH + CD + CV LiDAR parameters yielded the strongest correlations with canopy nitrogen concentration (CNC) (R = 0.35–0.79) and aboveground nitrogen accumulation (AGNA) (R = 0.85–0.92), values that markedly exceed those of any single- or dual-parameter combination (Table S2). In addition, these LiDAR parameters attained their highest correlations with CNC and AGNA at the 60 m flight height; for example, PH + CD + CV delivered R = 0.35–0.92 (60 m), surpassing 80 m (0.03–0.15) and 100 m (0.06–0.11) altitudes.

Table S2. correlation coefficient between nitrogen status and LiDAR based characteristic parameters.

Feature Parameters	Flight Altitude（m）	CNC		AGNA	
		R		R	
		VGS	FES	VGS	FES
PH	60	0.00	0.22	0.74	0.69
	80	0.00	0.20	0.64	055
	100	0.00	0.14	0.62	0.41
CD	60	0.00	0.46	0.81	0.82
	80	0.00	0.48	0.77	0.79
	100	0.00	0.46	0.71	0.77
CV	60	0.00	0.00	0.79	0.87
	80	0.00	0.00	0.74	0.81
	100	0.00	0.00	0.72	0.76
PH+CD	60	0.10	0.66	0.81	0.90
	80	0.00	0.62	0.75	0.85
	100	0.17	0.47	0.71	0.79
PH+CV	60	0.28	0.71	0.82	0.88
	80	0.14	0.22	0.78	0.82
	100	0.22	0.17	0.77	0.79
CD+CV	60	0.35	0.77	0.85	0.89
	80	0.17	0.72	0.82	0.87
	100	0.17	0.69	0.78	0.83
PH+CD+CV	60	0.35	0.79	0.85	0.92
	80	0.20	0.74	0.82	0.89
	100	0.24	0.69	0.79	0.84

Note: PH = plant height, CD = crown diameter, CV = canopy volume; hereinafter the same.

Multi-source feature importance and diagnostic analysis (Table S3) showed that the VIF for all fused parameters—including the optimized NDSI and LiDAR triplet—remained below 10. SHAP impact analysis revealed that while the optimized NDSI was the primary predictor for CNC (0.67–0.71), the contribution of structural metrics, specifically CV and CD, increased significantly for AGNA during the FES.

Table S3. Multicollinearity diagnostics (VIF) and feature importance analysis (SHAP) for the optimal LiDAR–HSI fusion models across phenological stages.

Target	Phenological Stage	Parameters	SHAP	VIF
CNC	VGS	NDSI (732, 879)	0.71	1.03
		PH	0.10	3.45
		CD	0.11	4.27
		CV	0.09	1.68
	FES	NDSI (560, 690)	0.67	1.97
		PH	0.13	1.20
		CD	0.14	4.20
		CV	0.06	2.73
AGNA	VGS	NDSI (711, 986)	0.67	2.26
		PH	0.10	6.12
		CD	0.13	6.89
		CV	0.06	3.29
	FES	NDSI (515, 736)	0.32	1.93
		PH	0.05	1.19
		CD	0.29	2.91
		CV	0.34	2.03

Table S4 compares model performance (R2) for CNC and AGNA under Uniform altitude and optimized altitude configurations. The optimized altitude consistently outperforms uniform altitude, yielding a 4.40%–8.99% accuracy improvement and a peak R^2 of 0.98. While uniform altitude offers operational simplicity, the optimized altitude strategy enhances precision by capturing complementary structural and spectral information.

Table S4. Comparison of model performance (R^2) under Uniform Altitude and Optimized Altitude for CNC and AGNA estimation.

Target	Phenological Stage	Uniform Altitude	Optimized Altitude
CNC	VGS	0.87±0.04	0.93±0.02
	FES	0.92±0.02	0.98±0.01
AGNA	VGS	0.89±0.03	0.97±0.01
	FES	0.91±0.02	0.95±0.02

Note: Uniform Altitude refers to both sensors operating at the same height: 100 m (LiDAR) and 100 m (Hyperspectral) for AGNA at VGS, and 60 m (LiDAR) and 60 m (Hyperspectral) for all other cases.
